# Supplementary material for: Crystallization and Structural Properties of Oleogel-Based Margarine
Source: Molecules. 2022 Dec 15;27(24):8952. doi: 10.3390/molecules27248952 (PMC9787469; doi:10.3390/molecules27248952)
Supplement: Supplementary file 1 [file molecules-27-08952-s001.zip › molecules-2051716-supplementary.pdf]

Supplementary data

Supplementary Table S1. Peak temperature (T_{pc}), and enthalpy (ΔH) of wax oleogel-based margarine with rapid cooling rate

Samples	T_{pc} (°C)	ΔH (Jg^{-1})
4%BW	$46.76±0.08^{a}$	$3.99±0.30^{a}$
6%BW	$49.13±0.28^{b}$	$4.30±0.19^{b}$
8%BW	$50.63±0.34^{c}$	$4.80±0.26^{c}$
10%BW	$52.23±0.23^{d}$	$9.51±0.54^{d}$
Commercial margarin	$33.45±0.31^{e}$	$4.03±0.30^{e}$

Values represent the average of three replicates ± standard deviation. Different superscript letters (a-e) denote significantly difference ($p < 0.05$) in the same column.

Supplementary Table S2. Peak temperature (T_{pc}), and enthalpy (ΔH) of wax oleogel-based margarine without rapid cooling rate

Samples	T_{pc}(°C)	ΔH(Jg^{-1})
4%BW'	$46.79±0.18^{a}$	$4.00±0.23^{a}$
6%BW'	$49.25±0.20^{b}$	$4.37±0.20^{b}$
8%BW'	$50.74±0.26^{c}$	$4.89±0.15^{c}$
10%BW'	$52.27±0.17^{d}$	$9.56±0.37^{d}$

Values represent the average of three replicates ± standard deviation. Different superscript letters (a-e) denote significantly difference ($p < 0.05$) in the same column.
